# Clodronate Liposome-Mediated Phagocytic Hemocyte Depletion Affects the Regeneration of the Cephalic Tentacle of the Invasive Snail, *Pomacea canaliculata*

**DOI:** 10.3390/biology12070992

**Published:** 2023-07-12

**Authors:** Giulia Bergamini, Sandro Sacchi, Anita Ferri, Nicola Franchi, Monica Montanari, Mohamad Ahmad, Chiara Losi, Milena Nasi, Marina Cocchi, Davide Malagoli

**Affiliations:** 1 Department Biology and Evolution of Marine Organisms, Zoological Station “Anton Dohrn”, 80121 Naples, Italy; giulia.bergamini@szn.it; 2 Department of Life Sciences, University of Modena and Reggio Emilia, 41125 Modena, Italy; sandro.sacchi@unimore.it (S.S.); nicola.franchi@unimore.it (N.F.); monica.montanari@unimore.it (M.M.); 283436@studenti.unimore.it (C.L.); 3 Department of Chemical and Geological Sciences, University of Modena and Reggio Emilia, 41125 Modena, Italy; 224420@studenti.unimore.it (A.F.); m.ahmad@live.nl (M.A.); marina.cocchi@unimore.it (M.C.); 4 LASIRE, Université de Lille, Cité Scientifique, 59650 Villeneuve-d’Ascq, France; 5 Department of Surgical, Medical and Dental Sciences, University of Modena and Reggio Emilia, 41125 Modena, Italy; milena.nasi@unimore.it; 6 NBFC, National Biodiversity Future Center, 90133 Palermo, Italy

**Keywords:** apple snail, mollusc, immunity, phagocytosis, inflammation, invertebrate, hemocyanin, transglutaminase, allograft inflammatory factor-1, image analysis

## Abstract

**Simple Summary:**

In the adult freshwater snail, *Pomacea canaliculata*, their cephalic sensory tentacles can regenerate after experimental amputation. Immediately after the amputation, the wound closes and a hemocyte-rich blastema is formed, but whether hemocytes play a role in blastema formation and the regeneration process is not known. Here, we have analyzed the effects of the chemical depletion of phagocytic hemocytes on tentacle regeneration. The transient depletion of phagocytic hemocytes was achieved by injecting the snails with clodronate liposomes before tentacle amputation. Flow cytometry demonstrated the effects of clodronate liposomes on molluscan phagocytic hemocytes. Histological analysis, followed by an application of an in-house developed semi-automated hemocyte count protocol, documented that in phagocytic hemocyte-depleted snails, the regeneration process was significantly delayed. When the number of circulating phagocytic hemocytes was restored to the control values, the regeneration process recovered. The expression in the blastema of genes associated with hemocyte-mediated functions, like oxygen transport, clotting and inflammation, was evaluated using RT-qPCR. Consistent with flow cytometry and histochemical observations, the blastema from clodronate liposome-injected snails, presented significantly modified gene expression levels, thus reinforcing the hypothesis of an active role of hemocytes in the onset of tentacle regeneration.

**Abstract:**

After amputation, granular hemocytes infiltrate the blastema of regenerating cephalic tentacles of the freshwater snail *Pomacea canaliculata*. Here, the circulating phagocytic hemocytes were chemically depleted by injecting the snails with clodronate liposomes, and the effects on the cephalic tentacle regeneration onset and on *Pc*-Hemocyanin, *Pc*-transglutaminase (*Pc*-TG) and *Pc*-Allograft Inflammatory Factor-1 (*Pc*-AIF-1) gene expressions were investigated. Flow cytometry analysis demonstrated that clodronate liposomes targeted large circulating hemocytes, resulting in a transient decrease in their number. Corresponding with the phagocyte depletion, tentacle regeneration onset was halted, and it resumed at the expected pace when clodronate liposome effects were no longer visible. In addition to the regeneration progress, the expressions of *Pc*-Hemocyanin, *Pc*-TG, and *Pc-*AIF-1, which are markers of hemocyte-mediated functions like oxygen transport and immunity, clotting, and inflammation, were modified. After the injection of clodronate liposomes, a specific computer-assisted image analysis protocol still evidenced the presence of granular hemocytes in the tentacle blastema. This is consistent with reports indicating the large and agranular hemocyte population as the most represented among the professional phagocytes of *P. canaliculata* and with the hypothesis that different hemocyte morphologies could exert diverse biological functions, as it has been observed in other invertebrates.

## 1. Introduction

Regeneration is a complex process that results in the complete regrowth of biological structures, ranging from single cells (e.g., axonal regrowth) to entire body components [1]. Regenerative capacity varies across the animal kingdom [2], and it is very limited in mammals [3]. Regeneration can be based on various events, from the rearrangement of pre-existing tissues in the absence of cell proliferation, to the proliferation of somatic stem cells or the dedifferentiation/transdifferentiation of cells that are in proximity or get to the wound [4]. The roles of various signaling pathways have been studied in many organisms [5], and overlaps have been found between developmental and regeneration events [4]. Considering the applications that could arise from the ability to promote controlled regeneration in human organisms, adult regeneration has been studied in numerous vertebrates and invertebrates [1,4,6]. The comparison between the regenerative capacities of vertebrates and invertebrates has highlighted the importance of the immune system [7]. Invertebrate models have an innate-only immune system, and numerous taxa show a significant ability to rebuild experimentally damaged components [8,9]. In this context, the regeneration of the *Pomacea canaliculata* cephalic tentacles has been documented. After the experimental amputation, the cephalic tentacles rapidly regrew, starting from a blastema that contained numerous granular hemocytes [10,11]. *P. canaliculata* is a freshwater snail that, due to its invasiveness and relevance to human health, has been the subject of molecular, behavioral, physiological, and immunological studies [12,13,14,15,16,17,18]. The immune system of *P. canaliculata* relies on both cellular and humoral components. Numerous studies have described the circulating hemocytes based on cell morphology [10,19,20], and a hemocyte-specific proteome has been released [12], but reliable hemocyte markers have not been indicated yet [21,22]. Advanced flow cytometry techniques have been used to classify the circulating hemocytes of *P. canaliculata* in the absence of hemocyte markers and have revealed a significant variety of circulating hemocyte populations [21].

Here, we tested the effects of a drug that specifically targets phagocytic circulating cells in both mammals and invertebrate models, i.e., chlodronate liposomes [23,24], and evaluated its influence on the circulating hemocyte number and the time course of the cephalic tentacle regeneration. Additionally, we performed RT-qPCR on the control regenerating tentacles and after clodronate liposome injection to analyze the gene expression profiles of *Pc*-Hemocyanin, *Pc*-transglutaminase *(Pc-*TG), and *Pc*-Allograft Inflammatory Factor -1 (*Pc-*AIF-1), which are potential markers of hemocyte-mediated functions, such as oxygen transport and immunity, clotting, and inflammation [25,26,27].

Our results indicate that *P. canaliculata* phagocytic hemocytes are successfully targeted by chlodronate liposomes and that their transient drop in number is associated with a delayed onset of tentacle regeneration and altered expression levels of the selected genes.

## 2. Materials and Methods

### 2.1. Snail Maintenance

The *Pomacea canaliculata* specimens were bred in the aquarium facility of the Department of Life Sciences, University of Modena and Reggio Emilia (Modena, Italy). The snails were housed were housed in static aerated aquaria at 25 ± 1 °C, 14:10 h light–dark photoperiod, with a maximal density of 1 snail/2 L of water [10]. Routine maintenance of aquaria included cleaning surfaces and water change (approximately 80% of the water), and it was performed twice a week. After the water change, snails were fed with a mix of green salad types. Experiments employed adult (6 to 10 months old, approximately 25–30 g of body weight), sexually mature, male and female snails.

### 2.2. Hemolymph Withdrawal

Diverse techniques of hemolymph withdrawal have been applied in *P. canaliculate* [10,28]. In this research, the hemolymph was collected by applying gentle pressure onto the operculum [10], thus allowing the repetition of withdrawals on the same animals [29]. The snails were starved for 3 to 5 days before the first hemolymph collection. Hemolymphs from each animal were allowed to be dropped directly into 1.5 mL tubes placed in granular ice [10] and immediately read using flow cytometry without adding anticoagulant or other chemicals. For microscopy observations, the protocol described in [10] was followed, and 1 volume of L-Cysteine (final concentration 1%) was added to prevent hemocyte clotting. Cytocentrifuged hemocytes were stained with BIO-DIFF kit (BioGnost^®^, Zagreb, Croatia). The hemolymphs were kept separated and never pooled.

### 2.3. Clodronate Liposome Treatment

A commercially available stock of clodronate liposomes (6.0 ± 0.5 mg/mL post reconstitution in molecular biology grade water) (Clophosome^TM^, Aurogene Srl, Rome, Italy) was injected with 1 mL sterile syringe in close proximity to the cephalic tentacle and the eye. The injection site was chosen because it has a maximal concentration of the drug in the amputation site, and it was maintained also for non-amputated snails. The snails were anesthetized before injection via immersion in granular ice for 30 min. After testing the supplier-recommended concentration, i.e., 200 µL for 20–25 g of animal body weight, the 0.1× and 10× concentrations were tested to assess their effects on hemocyte number and snail viability. On the basis of these preliminary observations, the selected concentration was 1× (i.e., approximately 45 μg/g snail) because it did not cause mortality while having significant impact on the circulating hemocyte number. After the injection, the animals were kept at room temperature (RT) for 30 min and then put into a freshly cleaned tank in ordinary maintenance conditions. Clodronate liposome exposure was fixed at 6, 24, and 48 h post-injection (hpi) timepoints. Explorative experiments with control empty liposomes suggested that the plain liposomes might affect hemocyte count, making it difficult to discriminate the effects of control liposomes in our treatments. Conversely, as hemocyte analysis from ice-anesthetized and saline-injected snails did not differ either from ice-anesthetized and uninjected snails or from non-anesthetized and uninjected snails, the latter was chosen as control in this research.

### 2.4. Flow Cytometry Analysis of Clodronate Liposome Effects

Acoustic-focused flow cytometry Attune NxT^®^ technology (Thermo Fisher Scientific, Waltham, MA, USA) prevents clogging and allowed a very fast analysis of snail circulating hemocytes, which were analyzed immediately after the withdrawal, with no additional treatment or staining. The number of events was fixed at 40,000 for each sample. Since, to our knowledge, no available information on clodronate liposome effects on *P. canaliculata* hemocytes was available, we opted to enclose all the events corresponding to diverse hemocyte populations [10] into a unique region of interest obtained from the SSC-FSC. For each snail, the R1 region obtained in control conditions before clodronate liposome injection was maintained for evaluating the hemolymph after the clodronate liposome injection. For each sample, the FSC-H vs. FSC-A parameter was applied to exclude that counted events, including cell aggregates.

### 2.5. Collection of Cephalic Tentacles for Histological and Gene Expression Analyses

To investigate the effects of clodronate liposomes on cephalic tentacle regeneration, non-injected snails (controls) and clodronate liposome-injected animals underwent tentacle amputation as previously described [30]. After amputation, snails were kept for 30 min at RT, then transferred into single cages placed in ordinary maintenance tanks for recovery. Regenerating tentacles of both untreated animals and clodronate liposome-injected animals were amputated again after 12, 24, or 48 h post-amputation (hpa). Three animals were used for each time point. Collected tentacles have been either fixed in freshly made Bouin’s solution for histological procedures [30] or lysed in 1 mL of TRI Reagent^®^ (Zymo Research; EuroClone, Milan, Italy) and stored at −80 °C before total RNA extraction and successive gene expression analysis.

### 2.6. Histological Analysis of Cephalic Tentacle Regeneration and Semi-Automatic Hemocyte Count

Cephalic tentacles from 3 control and 9 clodronate-liposome injected snails (N = 3 for each incubation interval) were fixed in freshly made Bouin’s solution for 6–8 h at RT then dehydrated by ascending scale of ethanol, starting from ethanol 70% to ethanol absolute by increasing ethanol percentage after every one-hour incubation. Samples were ultimately clarified with xylene and included in paraffin blocks. Slices of regenerating tentacles were obtained using manual microtome (7 μm thick sections) then moved onto degreased glass slide and dried overnight at 37 °C. Standard rehydration procedure was applied before hematoxylin–eosin staining. High-resolution digital micro-photographs of hematoxylin and eosin-stained tentacles were captured using EVOS M5000 Imaging System (ThermoFisher Scientific, Milan, Itay) mounting a 40× Olympus super-apochromat, coverslip-corrected AMEP4754 objective (NA: 0.95, diameter: 26.0, working distance: 0.18). Group II granular hemocytes were semi-automatically recognized and counted by applying an in-house developed protocol in MATLAB^®^ environment followed by multivariate image analysis (MIA), as described in detail elsewhere [30]. The validation step was performed in the previous publication [30], where a manual inspection was performed by an expert to visually check if the automated counting was appropriate.

### 2.7. Total RNA Extraction and Reverse Transcription (RT)

Stored samples from regenerating tentacles of non-injected or clodronate liposome-injected snails at 0, 12, and 24 hpa were processed for total RNA purification following the manufacturer’s protocol. RNA extraction from hemocytes was performed as described elsewhere [15]. After purification, total RNA was suspended in 20 μL of RNase-free water and then spectrophotometrically checked for quantity and quality using Nanodrop ND-1000 (Thermo Fisher Scientific, Waltham, MA, USA). One microgram of total RNA from each sample was reverse transcribed to cDNA using iScript cDNA synthesis kit (BioRad, Hercules, CA, USA), following the manufacturer’s protocol. The applied thermal profile consisted of 5 min at 25 °C, 20 min at 46 °C, and a final minute at 95 °C using a GeneAmp™ PCR System 2700 (Applied Biosystems, Waltham, MA, USA).

### 2.8. Primer Design

Primers were designed on mRNA sequence using the Primer3web version 4.1.0 [31,32] (Table S1).

As primer design parameters, the melting temperature was set between 58 °C and 62 °C, choosing 60 °C as optimal, primer length was set at 20 ± 2 base pair (bp), and amplicon size was set between 150 and 250 bp. Self- and cross-annealing were checked using the open-access tool available at http://www.operon.com/tools/oligo-analysis-tool.aspx link (last consulted on 15 May 2023). The genes *Pc*-Hemocyanin, *Pc*-Transglutaminase (TG), and *Pc*-Allograft inflammatory factor-1 (AIF-1) have been selected based either on their previous identification in circulating hemocytes of *P. canaliculata* via LC-MS/MS [33] or our preliminary unpublished observations as proteins involved in multiple immunological functions. Ribosomal protein L5 (RpL5) gene has been used as reference gene for *P. canaliculata* since its expression is stable in many tissues [13,15]. Primers were designed to amplify partial sequence encoding for conserved functional domains of their protein as demonstrated via NCBI Conserved Domain Search and Conserved Domain Architecture Retrieval Tool, free bioinformatic tools of National Center for Biotechnology Information (NCBI). Designed primers were finally checked with Basic Local Alignment Search Tool nucleotide-nucleotide (BLASTn^®^) and translated nucleotide-protein BLASTx^®^ to ensure specificity. RT-PCR experiments were performed for qualitative evaluation of the basal expression of the selected genes in circulating hemocytes (Figure S1), before proceeding further with quantitative experiments on regenerating cephalic tentacles.

### 2.9. RT-Quantitative PCR (RT-qPCR)

The cDNAs (1 μL) from regenerating tentacles of uninjected control or clodronate liposome-treated snails were employed as templates in RT-qPCR reactions using SsoAdvanced Universal SYBR^®^ Green Supermix (BioRad, Hercules, CA, USA). Primers were added to the reaction mix at the final concentration of 250 nM. Single samples were run in triplicate, and their expression levels were reported as mean average for the successive gene expression analysis. qPCR reactions were conducted on BioRad CFXconnect Real Time System (BioRad, Hercules, CA, USA) applying the same thermal profile for all the evaluated genes: 30 s at 95 °C, 40 cycles of 5 s at 95 °C and 20 s at 60 °C. Results were normalized using RpL5 as reference gene. Negative control reactions were performed in triplicate using 1 μL of RNase-free water instead of cDNA as template. The specificity of each reaction was evaluated by running amplicons via a 1.2% agarose gel in 1× standard Tris-Boric acid-EDTA (TBE) buffer and by carefully observing the dissociation curve provided by the instrument. Gene expression was analyzed by applying the 2^−ΔΔCt^ method within the free software Past^®^.

### 2.10. Statistical Analysis

Flow cytometry-based hemocyte count in the region of interest before and after clodronate injection was analyzed by applying Dunnett’s test as appropriate (*p* < 0.05), set for statistical significance.

Results from semi-automated count have been processed according to the Tukey–Kramer test (*p* < 0.05).

For qPCR results, Shapiro–Wilk and homoscedasticity F test were first applied to assess specimens’ homoskedasticity (*p* > 0.05), then qPCR data underwent logarithmic transformation (log_2_(2^ΔCttarget/2ΔCtRpl5^), and were analyzed with either a paired *t*-test or Wilcoxon test.

## 3. Results

### 3.1. Clodronate Liposomes Transiently Reduced the Number of Large Circulating Hemocytes

**Figure 1:**
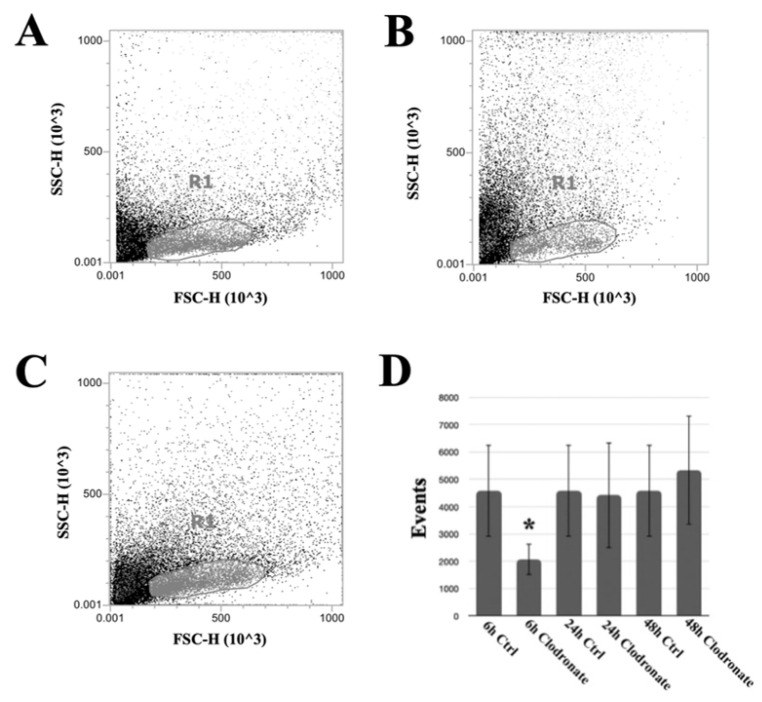
Clodronate liposome effects on circulating hemocytes of *P. canaliculata*. For each condition, 1 animal representative of a group of 10 is represented. Flow cytometry-based analysis of (**A**) control hemolymph, (**B**) 6 h after the injection of clodronate liposomes, and (**C**) 24 h after the injection of clodronate liposomes. The R1 gate included all the circulating hemocytes, excluding debris collected with the hemolymph. Clodronate liposome effects on circulating hemocytes could be observed only at 6 h after the injection and mainly on cells with a higher FSC value (**B**). (**D**) Number of events gated within R1 region at different timepoints. * *p* < 0,05 according to Dunnett’s test. The microscopical analysis of hemocytes from clodronate liposome-injected snails indicated an increase in the relative presence of small/intermediate hemocytes (Figure S2).

Acoustic focusing flow cytometry was applied in order to quantitatively investigate the effects of the phagocyte-targeting clodronate liposomes. The hemolymph collected from non-injected and from clodronate-injected *P. canaliculata* individuals presented a significant difference at 6 h after the injection (Figure 1). More in detail, the number of events corresponding to the *P. canaliculata* hemocytes with a higher FSC value was significantly reduced at 6 h after the injection of clodronate liposomes (Figure 1A). Twenty four h after the clodronate liposome injection, the recovery in the depleted hemocyte population was visible (Figure 1A–C), and it was maintained at 48 h after the injection (Figure 1D).

### 3.2. Granular Hemocytes were still Recognized in Regenerating Tentacles after Clodronate Liposome Injection

**Figure 2:**
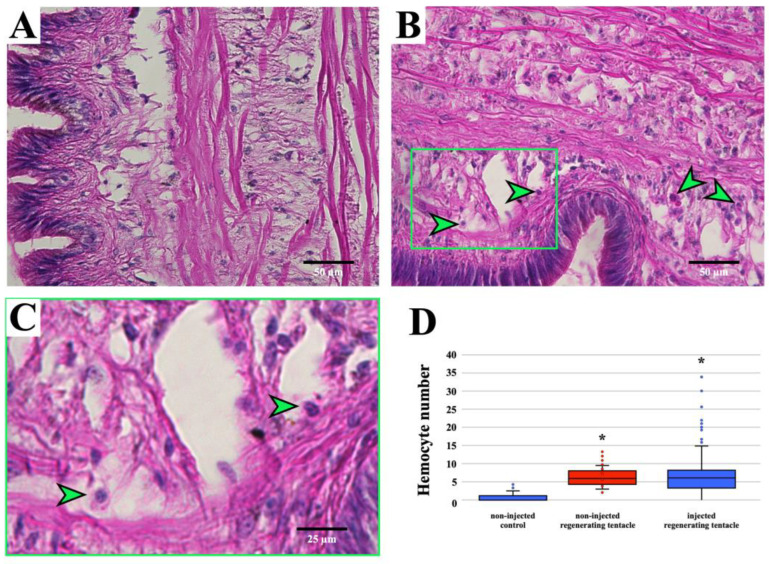
Automated granular hemocyte identification and count using the in-house routine. (**A**) non-regenerating and (**B**) clodronate liposome-injected regenerating tentacles at 12 hpa. Examples of cells recognized as large and granular hemocytes are indicated (green arrowheads). (**C**) Magnified image representing a detail of Figure (**B**). Examples of cells with a morphology recognized by the computer-assisted hemocyte count are indicated by green arrowheads. During the setup of the in-house developed protocol in MATLAB^®^ environment, the validation step was performed via a manual inspection, to visually check if the automated counting was appropriate [30]. (**D**) Computer-assisted hemocyte count in non-regenerating control, regenerating tentacles (12 hpa), and clodronate liposome-injected regenerating tentacles (12 hpa). Data from regenerating tentacles (12 hpa) have already been presented in a previous publication [11]; hence, they arereported in a different color. * *p* < 0.05 according to the Tukey–Kramer test.

The in-house developed routine (in MATLAB^®^ environment) semi-automated granular hemocyte count was performed on images collected from the histological sections of the regenerating cephalic tentacle allowing for the quantification of granular hemocytes in the blastema 12 h post-amputation (hpa), which was the first time point with enough tissue for the quantitative analysis. At 12 hpa, granular hemocytes were present, and their number in the histological sections from clodronate liposome-injected snails was significantly higher than the non-regenerating tentacle (0 hpa), whereas it did not differ significantly from the data collected in the previous experiments (Figure 2).

### 3.3. Clodronate Liposomes Affected the Timing of Cephalic Tentacle Regeneration

**Figure 3:**
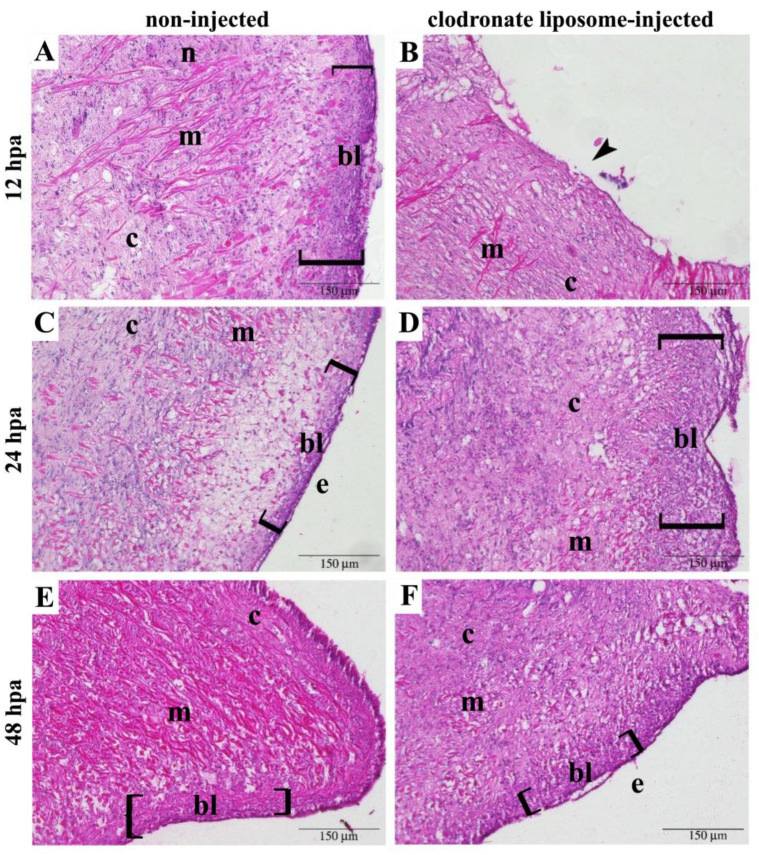
Wound closure and blastema formation were delayed steps in the tentacle regeneration of clodronate liposome-injected *P. canaliculata*. For each experimental condition and time, 1 animal representative of a group of 3 is represented. Non-injected snails showed (**A**) a well-recognizable blastema (bl), (**C**) a newly formed epithelium (e) on the wound surface, and (**E**) a smaller blastema under the re-epithelialized wound (brackets) at 12, 24, and 48 hpa, respectively. Clodronate liposome-injected animals, (**B**) still presented an open wound (arrowhead) at 12 hpa. (**D**) The blastema (bl) was well recognizable at 24 hpa (brackets), under a closed but not completely re-epithelialized wound. (**F**) Re-epithelialization (e) was completed at 48 hpa. Abbreviations: connective tissue (c), muscle (m), nerve (n). Magnified details of the wounds represented in each panel are presented in Figure S3.

A different time course was observed in the response to the experimental amputation between non-injected and clodronate liposome-treated snails (Figure 3). Non-injected specimens displayed an evident blastema at 12 hpa, the epithelium delimiting the closed wound surface at 24 hpa, and a smaller blastema at 48 hpa (Figure 3). The histological organization of these components was not altered after clodronate liposome injection, but their onset was delayed. Consequently, in clodronate-injected snails at 12 hpa, the wound margin was still open, and no blastema was visible (Figure 3A and Figure S3); at 24 hpa, the blastema became evident (Figure 3B and Figure S3); and at 48 hpa, the wound margin was closed, and a new epithelium was evident (Figure 3C). Although tentacle regeneration was delayed after clodronate liposome injection, the microscopic appearance of the regenerating tentacle and of the tissues in close proximity to the wounded area was similar, and the time course was the only difference that could be observed between the regenerating tentacles of the control and clodronate liposome-injected snails.

### 3.4. Clodronate Liposomes Affected the Expression of Hemocyte-associated Genes

**Figure 4:**
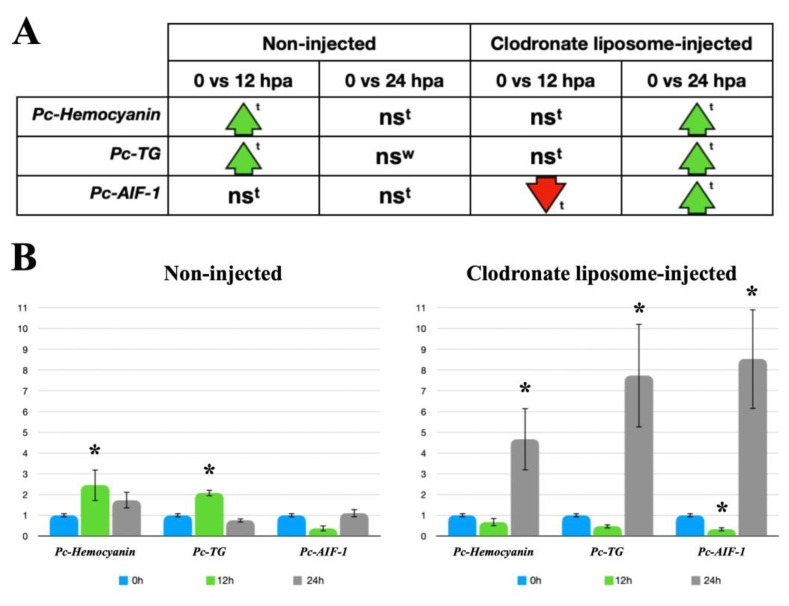
RT-qPCR analysis of *Pc*-Hemocyanin, *Pc*-TG, and *Pc*-AIF-1 expressions in regenerating tentacles of *P. canaliculata.* (**A**) Schematic representation of RT-qPCR results: ns: no significant difference, ↑: significant increase; ↓: significant decrease. Data were analyzed with either Student’s *t*-test (^t^) or Wilcoxon test (^W^) (*p* < 0.05). (**B**) Left: *Pc*-Hemocyanin, *Pc*-TG, and *Pc*-AIF-1 basal (0 h) and amputation-modified expressions (12 and 24 h) in regenerating tentacles. (**B**) Right: *Pc*-Hemocyanin, *Pc*-TG, and *Pc*-AIF-1 basal (0 h) and post-amputation expressions (12 and 24 h) in regenerating tentacles from either control (non-injected) or clodronate liposome-injected snails. * *p* < 0.05 according to Student’s *t*-test.

*Pc*-Hemocyanin, *Pc*-TG, and *Pc*-AIF-1 were selected for qPCR analysis as potential indicators of hemocyte-related functions. Their expressions were qualitatively evaluated in circulating hemocytes using RT-PCR (Figure S1) and then quantitatively studied in non-regenerating tentacles via RT-qPCR to determine the constitutive levels of expression, as a reference point (0 hpa). Subsequently, the expressions of *Pc*-Hemocyanin, *Pc*-TG, and *Pc*-AIF-1 were investigated in the regenerating tentacles of snails that were either not injected or injected with clodronate liposomes at 12 and 24 hpa. Compared to 0 hpa, qPCR revealed a significant increase in *Pc*-Hemocyanin and *Pc*-TG expression in non-injected snails, at 12 hpa, with expression levels returning to 0 hpa levels at 24 hpa. There were no significant changes in *Pc*-AIF-1 expression between non-regenerating and regenerating tentacles from non-injected snails at either 12 or 24 hpa (Figure 4). In clodronate liposome-injected snails, Pc-Hemocyanin and *Pc*-TG expressions were similar to control levels at 12 hpa but significantly higher at 24 hpa. For *Pc*-AIF-1, a significantly reduced expression was observed at 12 hpa, and augmented levels were measured at 24 hpa (Figure 4).

## 4. Discussion

*P. canaliculata* circulating hemocyte populations have been categorized and referred to by different names [10,19,28]. Therefore, the terminology proposed for *P. canaliculata* after applying the Image3C method, which is derived from an automated classification of cells using convolutional neural networks and avoiding user bias or manual classification, will be utilized here [21]. Components within the pericardial cavity and the hemocyte islets of the kidney have been indicated as potential hematopoietic sites [20,29], and a circulating hemocyte subpopulation has also been proposed as a possible source of new hemocytes [30]. The genome of *P. canaliculata* has been released [17,31], and the proteome of the whole circulating hemocyte population has been sequenced [14,22]. The lack of genetic tools and hemocytic molecular markers [21,22] highlights the need for further experiments to better define the hemocyte functions in immune and non-immune biological contexts.

Clodronate cell-permeable liposomes have been successfully utilized in vertebrates [24,32,33] and invertebrates [23,34,35] to obtain a chemical depletion of phagocytic cells. In adult females of the mosquito *Anopheles gambiae*, the administration of clodronate liposomes, followed by flow cytometry, microscopy, and molecular analyses, allowed for the identification of at least three phagocytic cell types and contributed to shedding new light on the immune response mediated by phagocytic immune cell subtypes against *Plasmodium* infection [35]. Therefore, the effects of clodronate liposome injections on *P. canaliculata* phagocytic cells were investigated using combined morphology- and molecular-based approaches.

As flow cytometry analysis of withdrawn hemocytes provided slightly discordant results based on the withdrawal methods and reagents applied [16], the flow cytometry analysis of the effects of clodronate liposomes on *P. canaliculata* circulating hemocytes was focused on the whole cell population. A constant match between the events corresponding to the whole cell population and the FSC-H vs. FSC-A dot plot allowed us to exclude that the counted events corresponded to cell aggregates. In agreement with the flow cytometry data, a parallel morphological analysis of hemolymph from clodronate liposome-treated snails indicated an increase in the relative abundance of small/intermediate hemocytes [10,19,21,28]. Clodronate liposomes significantly reduced the number of circulating hemocytes, especially targeting larger hemocytes, consistent with the conclusions derived via the application of Image3C, indicating the agranular large hemocytes as the main representative professional phagocytes in *P. canaliculata* [21]. The effects of clodronate liposomes were transient, and within 24 h, the circulating hemocyte number and populations were restored, as shown by the flow cytometry analysis. This evidence supports the hypothesis about the existence of hemocyte reservoirs in *P. canaliculata* [20,29]. However, at this stage, the replication or maturation of the remaining circulating hemocytes, i.e., mainly the smaller hemocytes on the basis of flow cytometry and morphological analyses, cannot be excluded [10,21,30].

The involvement of circulating hemocytes in cell-mediated immune responses and the synthesis of immune-related molecules has already been confirmed in *P. canaliculata* [13,15,21,28,36]. As the number of physiological responses regulated by, or associated with, immune-related cells and molecules is increasing [37], the focus of subsequent experiments has been to investigate whether the chemical depletion of phagocytic immune-related cells could affect complex organ regeneration, in the absence of a specific immune challenge [11,38]. In previous experiments, it was demonstrated that adult *P. canaliculata* could regenerate the cephalic tentacle, and the presence of granular and large hemocytes therein was quantified via an in-house developed routine for semi-automated computer-assisted image analysis [11]. The granular large hemocytes were semi-automatically recognizable for their specific stainability, whereas the other hemocytes could not be distinguished via image analysis from the other cells present in the histological sections. The presence of granular large hemocytes increased from 5 to 9 times in the blastema and the surrounding tissues within 12 h post-amputation [11]. Hemocyte accumulation was no longer visible 24 h after the amputation when the blastema was also reduced in size [11]. In present experiments, after clodronate liposome injection, semi-automated computer-assisted image analysis still evidenced a significant increase in large and granular hemocytes in the regenerating tentacle, consistent with the observation that the most represented population among the professional phagocytic hemocytes, i.e., the target of clodronate liposomes, is the large and agranular hemocytes [21]. In this context, it has to be remarked that also granular hemocytes have been indicated as phagocytic cells [21,28], although to a lesser extent than large and agranular hemocytes. This might justify the slightly lower number of large and granular hemocytes registered in regenerating cephalic tentacles from clodronate liposome-injected snails with respect to previous experiments [11]. However, since individual and sex-based differences have been registered for large and granular hemocyte numbers [21], modest differences in the abundance of these cells among diverse experimental sets should be considered carefully for their biological significance.

The histochemical analysis of the regenerating cephalic tentacles in snails injected with clodronate liposomes demonstrated that the depletion of circulating large hemocytes is concomitant with the slowing down of the regenerating process. The comparison between non-injected and clodronate liposome-injected snails evidenced delayed blastema organization and wound re-epithelization, which were observed at 24 and 48 hpa, respectively, in injected snails, instead of 12 and 24 hpa as in non-injected snails. The regeneration process in clodronate liposome-injected snails progressively recovered, and at 72 hpa, no evident histological differences could be highlighted using morphological staining and light microscopy. This observation allowed us to hypothesize that phagocytic cells may be necessary for the proper onset of cephalic tentacle regeneration in *P. canaliculata*. The molecular dialogue between immune components and the extracellular matrix is considered one of the key aspects of the regenerative process in all metazoans [7]. The importance of phagocytic cells in complex tissue regeneration has been reported in vertebrates, where macrophages, cells bridging innate and adaptive immune responses, have repeatedly been reported as key players. In axolotl, the injection of clodronate immediately before the injury has been associated with regenerative limb defects [39], whereas clodronate injection after blastema formation did not significantly impact limb regeneration [39]. Similarly, the clodronate liposome-mediated pre-depletion of macrophages resulted in a compromised regeneration of the heart in zebrafish, demonstrating that the timely recruitment of macrophages was necessary during heart regeneration in *Danio rerio* [40]. In regenerating vertebrates, the cytokine network and the balancing among different immune cell populations have been indicated as key components for the successful regeneration of complex tissues [41]. The vast majority of immune systems in invertebrate organisms are understudied, and only a few cytokines have been identified in molluscs [42]. Nonetheless, phagocytic cells seem crucial for regeneration in invertebrates as well as vertebrates. In the cricket *Gryllus bimaculatus*, the chemical depletion of phagocytic cells (i.e., plasmatocytes), via clodronate liposomes, negatively affected leg regeneration [38]. Microglial cells are necessary for the regeneration and repair of injured neural components in the Annelid *Hirudo medicinalis* [43,44]. In gastropods the activation of phagocytic cells and the regulation of the cell-mediated immune response have been indicated as crucial components in the regeneration of neural components [45]. Based on these findings, the correlation between phagocyte depletion and delayed onset of tentacle regeneration was further investigated on a molecular basis. No hemocyte markers are available for *P. canaliculata* circulating hemocytes [22], but hemocyte-derived proteins have been identified [14]. Here, we investigated the expressions of *Pc*-Hemocyanin, *Pc*-TG, and *Pc*-AIF-1. *Pc*-Hemocyanin is a multimeric protein that has been studied in molecular detail [25]. Similar to other molluscs [46,47,48], *Pc*-Hemocyanin could have immune-related roles besides oxygen binding. In terms of expression sites or cells, *Pc*-Hemocyanin has been retrieved in the albumen gland transcriptome [49] and hemocyte proteome [14]. The processes leading from the synthesis to the assembly of *Pc*-Hemocyanin have not been clarified yet, although the involvement of rhogocytes and the kidney has been hypothesized [16]. During the first phase of tentacle regeneration, RT-qPCR experiments have demonstrated a significant increase in *Pc*-Hemocyanin expression. This increase was transient and returned to non-regenerating tentacle levels after 24 h from the injury. In phagocyte-depleted snails, the expression of *Pc*-Hemocyanin followed the profile of tentacle regeneration progress, with a significant increase at 24 h after the amputation instead of 12 h. Similarly, *Pc*-TG expression was significantly upregulated at 12 hpa in regenerating tentacles of non-injected snails, but the upregulation was delayed at 24 hpa in clodronate-liposome-injected snails. Studies have shown that TG acts as an immune-related coagulant protein in *Crassostrea gigas* exposed to bacterial challenge; its expression is unaffected in hemocytes exposed to *Vibrio campbellii*, while it increases in oysters exposed to four pathogenic *Vibrio* strains [26,50]. Hemocytes in *Patinopecten yessoensis* produce a specific TG that may intervene upon tissue injury [51]. In the shrimp *Litopenaeus vannamei*, a significant interaction has been proposed between hemocyanin and TG, modulating its expression and affecting clotting upon pathogen exposure [52]. Since, to our knowledge, this interaction has not been reported in molluscs, and although our data on gene expression suggest it might occur in *P. canaliculata*, further studies are required to explore this possibility. AIF-1 is a molecule with an immunomodulatory role in mammals, and it is also widely distributed in invertebrates, where it is mainly associated with inflammation [27]. In molluscs, AIF-1 expression has been reported to increase after immune challenges, tissue injury, and pollution [53,54,55]. In bivalves, AIF-1 has been reported to act as a pro-inflammatory cytokine, maximally expressed in hemocytes [55,56] and associated with inflammatory reactions [57,58]. In non-injected snails, *Pc*-AIF-1 expression was not affected during the onset of tentacle regeneration, as RT-qPCR experiments did not evidence significant changes, in comparison with non-regenerating tentacles. Conversely, *Pc*-AIF-1 was downregulated in clodronate liposome-injected snails at 12 hpa, whereas at 24 hpa, its expression levels were significantly higher than those registered for non-regenerating tentacles.

Previous experiments have documented the presence of hemocytes during the first phases of regeneration of the cephalic tentacle in *P. canaliculata* [11]. Here, by combining flow cytometry and histological and molecular data, it can be hypothesized that phagocytic hemocytes are necessary for the early onset of tentacle regeneration. The clodronate liposome-mediated depletion of circulating hemocytes was associated with the delayed start of regeneration. When the population of circulating hemocytes was recovered, the tentacle regeneration proceeded without histological alterations or structural defects. In order to strengthen the correlation between the circulating hemocyte population profile and the tentacle regeneration timing, we explored the expression of hemocyte- and immune-related molecules using RT-qPCR. In non-regenerating tentacles, the basal expression of *Pc*-hemocyanin, *Pc*-TG, and *Pc*-AIF-1 has been demonstrated. The expression levels of *Pc*-hemocyanin and *Pc*-TG increased soon after tentacle amputation in non-injected snails, when the blastema was forming, and they were again similar to controls within 24 h, when the wound was closed and re-epithelized, and the regeneration process had started. However, *Pc*-AIF-1 expression did not change significantly during the first 24 h of regeneration. While the function of *Pc*-AIF-1 and its potential involvement in *P. canaliculata* regeneration requires further investigation, it has to be highlighted that in axolotls, one of the most significant regeneration models in vertebrates, molecular evidence suggests an early accumulation of inflammation-resolving macrophages into the blastema [59]. Although knowledge about the *P. canaliculata* hemocyte functions is currently limited, the complexity revealed in the circulating populations [21] does not exclude the possibility that the hemocyte recruitment and/or activation at a wounded site may be specific also in *P. canaliculata*. In these regards, snails injected with clodronate liposomes failed to initiate proper wound healing and tentacle regeneration, suggesting that phagocytic hemocyte depletion may have impacted regeneration, as observed in other models [38]. Moreover, RT-qPCR experiments in clodronate liposome-injected snails showed an upregulation of *Pc*-hemocyanin and *Pc*-TG only at 24 hpa, in association with circulating hemocyte recovery and wound closure. Additionally, since the number of large and granular hemocytes in amputated tentacles after the injection of clodronate liposomes remained higher than in non-regenerating tentacles, it is conceivable that this hemocyte population alone is insufficient for a proper onset of tentacle regeneration.

## 5. Conclusions

On the whole, our data indicated in clodronate liposomes a useful mean for studying hemocyte depletion effects in the gastropod *P. canaliculata* and highlighted a biological context, i.e., cephalic tentacle regeneration, suitable to explore the biological roles of the numerous hemocyte populations of this snail, also in absence of a direct immune challenge [60,61,62,63].

## Data Availability

Not applicable.

## Supplementary Materials

The following supporting information can be downloaded at: https://www.mdpi.com/article/10.3390/biology12070992/s1, Figure S1: Expression of *Pc*-Hemocyanin, *Pc*-TG, and *Pc*-AIF-1 in circulating hemocytes. Figure S2: Microscopical analysis of hemocytes from clodronate liposome-injected snails. Figure S3: Clodronate liposome injection delayed wound closure and blastema formation during the cephalic tentacle regeneration in *P. canaliculata* (magnified details); Table S1: Primer List.
