# Supplementary material for: Clodronate Liposome-Mediated Phagocytic Hemocyte Depletion Affects the Regeneration of the Cephalic Tentacle of the Invasive Snail, Pomacea canaliculata
Source: Biology (Basel). 2023 Jul 12;12(7):992. doi: 10.3390/biology12070992 (PMC10376890; doi:10.3390/biology12070992)
Supplement: Supplementary file 1 [file biology-12-00992-s001.zip › Table S1.pdf]

Table S1 Primer List

Primer Name	Sequence 5'-3'	Amplicon size (bp)	Target Gene Name	Accession #
Pc-Hemocyanin_F	TCACCCACAACGCC ATCCAC	228	hemocyanin G-type, units Oda to Odg-like (LOC112561353)	XM_025233 779.1
Pc-Hemocyanin_R	TGCTGTCCCAGTTG AAGGGC			
Pc-TG_F	AGTCGCAATGTGAA CCGCCA	140	protein-glutamine gamma-glutamyltransferase K-like (LOC112576690)	XM_025259 333.1
Pc-TG_R	TGAGCACGATGCTG TCGTCG			
Pc-AIF-1_F	GCTACTGGCGCAAA GCCTAA	213	allograft inflammatory factor 1-like (LOC112566076)	XM_025242 021.1
Pc-AIF-1_R	TTGTGGCAGTTCCTC ATCAC			
RPL5_F	CGTATGCCAGAATT GAGGGT	252	60S ribosomal protein L5-like (LOC112575270)	XM_025256 997.1
RPL5_R	CAACATCCAAGTAT GCACGG			
