# Supplementary figures and images for: Clodronate Liposome-Mediated Phagocytic Hemocyte Depletion Affects the Regeneration of the Cephalic Tentacle of the Invasive Snail, Pomacea canaliculata
Source: Biology (Basel). 2023 Jul 12;12(7):992. doi: 10.3390/biology12070992 (PMC10376890; doi:10.3390/biology12070992)

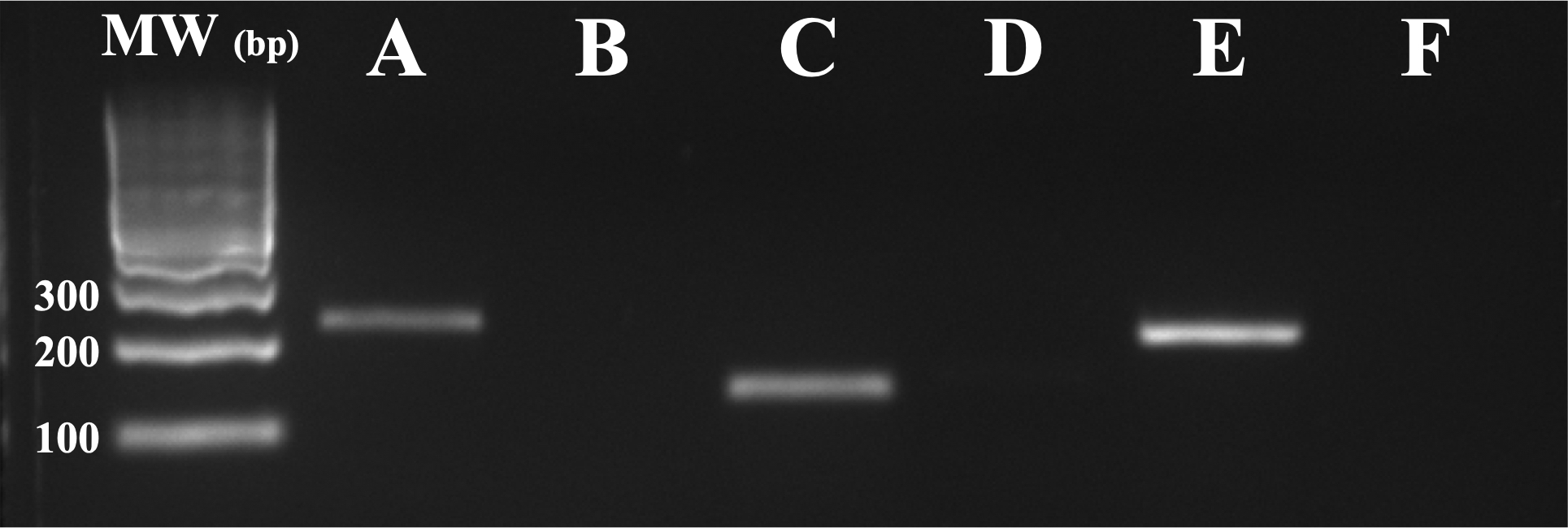

Supplement: Supplementary file 1 [file biology-12-00992-s001.zip › Fig_S1.jpg]

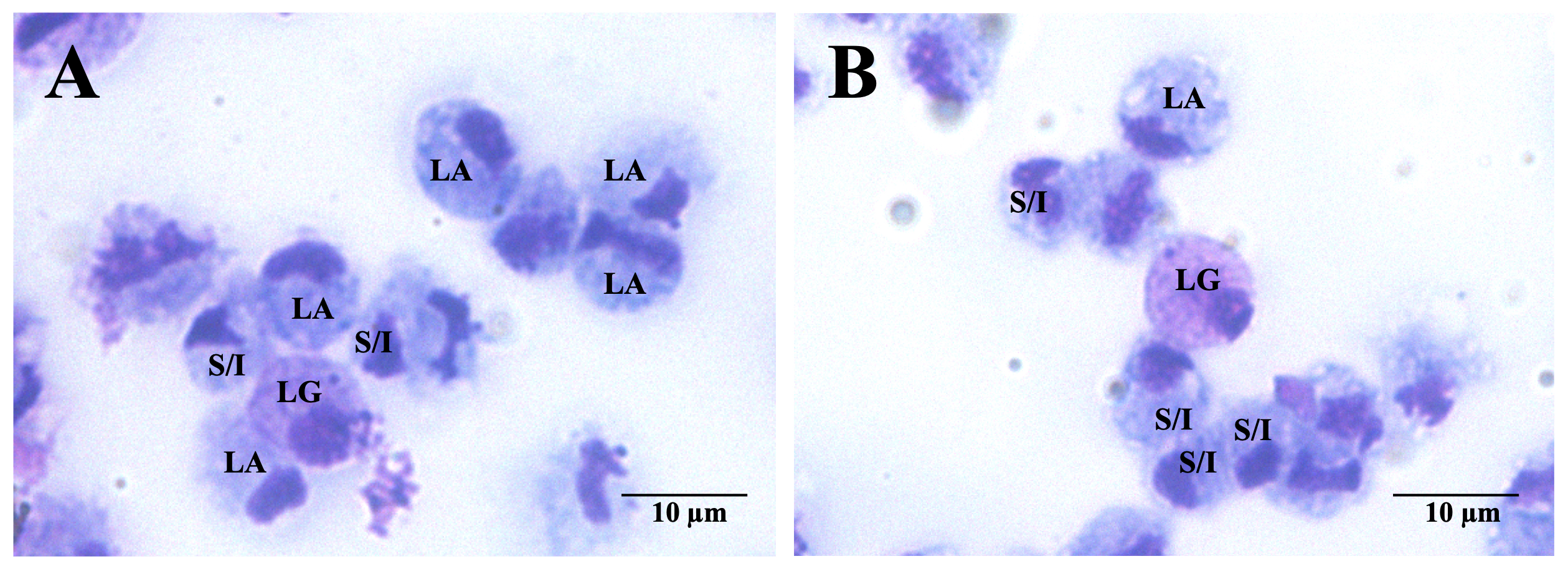

Supplement: Supplementary file 1 [file biology-12-00992-s001.zip › Fig_S2.jpg]

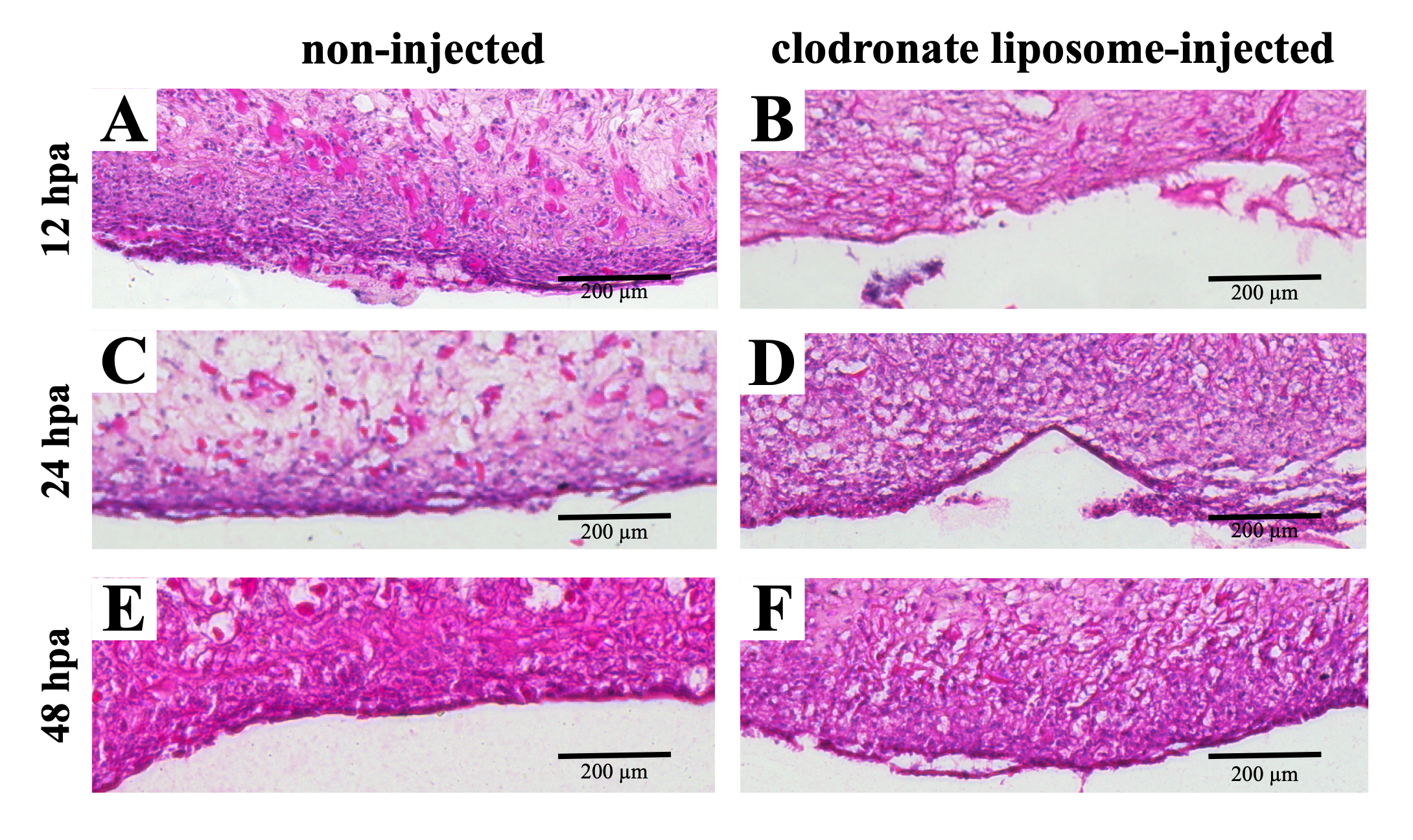

Supplement: Supplementary file 1 [file biology-12-00992-s001.zip › Fig_S3.jpg]
